# An Update on the Potential of Mesenchymal Stem Cell Therapy for Cutaneous Diseases

**DOI:** 10.1155/2021/8834590

**Published:** 2021-01-05

**Authors:** Yanyun Li, Ziyu Ye, Weiqin Yang, Qunzhou Zhang, Jincheng Zeng

**Affiliations:** ^1^Guangdong Provincial Key Laboratory of Medical Molecular Diagnostics, Dongguan Key Laboratory of Medical Bioactive Molecular Developmental and Translational Research, Guangdong Medical University, Dongguan 523808, China; ^2^Department of Oral and Maxillofacial Surgery and Pharmacology, University of Pennsylvania School of Dental Medicine, Philadelphia 19104, USA

## Abstract

Mesenchymal stem or stromal cells (MSCs) are nonhematopoietic postnatal stem cells with self-renewal, multipotent differentiation, and potent immunomodulatory and anti-inflammatory capabilities, thus playing an important role in tissue repair and regeneration. Numerous clinical and preclinical studies have demonstrated the potential application of MSCs in the treatment of tissue inflammation and immune diseases, including inflammatory skin diseases. Therefore, understanding the biological and immunological characteristics of MSCs is important to standardize and optimize MSC-based regenerative therapy. In this review, we highlight the mechanisms underlying MSC-mediated immunomodulation and tissue repair/regeneration and present the latest development of MSC-based clinical trials on cutaneous diseases.

## 1. Introduction

Mesenchymal stem cells (MSCs) represent a unique population of postnatal multipotent stem cells existing in a variety of adult, perinatal, and fetal tissues [1]. Although the existence of MSCs was proposed by Cohnheim about 150 years ago, they were definitely identified in the bone marrow and designated as bone marrow stromal stem cells (BMSCs) by Friedenstein in the 1970s [2]. Subsequently, it has been further demonstrated that MSCs are immune privileged and possess potent self-renewal, multipotent differentiation, and immunomodulatory/anti-inflammatory capabilities [3]. Due to these unique properties of MSCs, they have been widely explored as a potential cell-based regenerative therapy for a large spectrum of diseases, such as myocardial infarction [4], inflammatory bowel disease [5], cancer [6], glaucoma [7], osteoarthritis [8], nervous system disorders [9], and oral and maxillofacial diseases [10]. Skin disorders caused by aging, various types of environmental and genetic factors, trauma, and systemic diseases, e.g., diabetes and graft versus host disease (GVHD), represent one of the major public health burdens worldwide that significantly affect the quality of life of patients [11]. Currently, there are limited treatment options for these dermatological disorders due to the complicated causes and our limited understanding of the mechanisms underlying their pathogenesis [11]. In the last decade, MSC-based therapy is emerging as a novel paradigm for the treatment of various skin disorders [12]. In this review, we will discuss our current understanding of the function and mechanism of actions as well as the potential application of MSCs in the treatment of cutaneous diseases.

## 2. Characterization of MSCs

**Figure 1:**
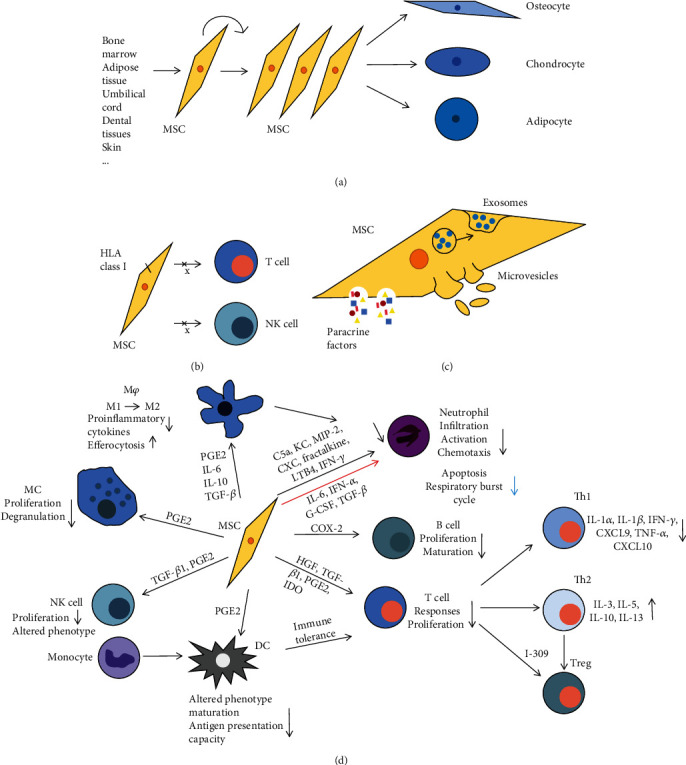
Schematic representation of the mechanisms of MSCs to promote tissue repair and immunosuppression. (a) Self-renewal and multipotent differentiation functions. (b) Low immunogenicity. (c) Transfer of biologically active substances by paracrine or EVs. (d) Immunomodulatory properties.

MSCs can be easily isolated from human donors and rapidly expanded in vitro without loss of their main biological properties [13]. Up to date, MSCs have been isolated from various types of tissues, such as the bone marrow [14], adipose tissue [14], skeletal muscles [14], synovium [15], dental pulp [15], placenta [15], umbilical cord blood [15], umbilical cord [15], gingiva [16], amnion [17], umbilical cord Wharton's jelly [18], and skin [14, 15]. However, large variations exist in the quality and biological function of MSCs of different tissue origins due to the subtle differences in the isolation and ex vivo culture and expansion and the lack of consistent markers for MSC identification. To solve this issue, the Mesenchymal and Tissue Stem Cell Committee of the ISCT has proposed minimal criteria to define human MSCs: (a) plastic adherent; (b) the positive expression of CD105, CD73, and CD90 and the lack of expression of hematopoietic cell markers, e.g., CD45, CD34, CD14 or CD11b, CD79a or CD19, and HLA class II; and (c) the differentiation capability into osteoblasts, adipocytes, and chondroblasts in vitro [19, 20] (Figure 1(a)). In addition to these basic properties, MSCs possess potent self-renewal and immunomodulatory/anti-inflammatory functions but are immune privileged due to their expression of a high level of HLA class I molecule, the absent expression of HLA class II, and the T cell costimulatory molecules B7-1, B7-2, CD40, or CD40L [21], thus making MSCs tolerable to allogeneic T cells [22] and freshly isolated allogeneic natural killer (NK) cells [23] (Figure 1(b)). However, the expression of HLA class II molecule could be induced in undifferentiated MSC by treatment with IFN-*γ* or during MSC differentiation [21]. Therefore, both the autologous and allogeneic MSCs could be utilized for cell-based therapy in regenerative medicine.

## 3. Immunomodulatory Properties of MSCs

MSCs have potent immunomodulatory effects on various types of innate and adaptive immune cells (Figure 1(d)). Among innate immune cells, MSCs of different tissue origins are capable of inhibiting the activation of proinflammatory M1 macrophages and promoting the polarization of macrophages toward anti-inflammatory M2 phenotypes as evidenced by decreased production of proinflammatory cytokines, increased secretion of anti-inflammatory mediators, and enhanced efferocytosis of apoptotic cells [24–27]. A recent study indicated that MSC-mediated reduction of CXC chemokines was achieved via enhancing the intracellular activation of p38 MAPK phosphorylation and inhibiting the NF-kappaB p65 phosphorylation in macrophages [28]. In terms of NK cells, MSCs alter their phenotypes and inhibit their proliferation and cytokine secretion either by cell-cell contact or through the mediation of soluble factors, including TGF-*β*1 and PGE2 [23]. In addition, MSCs regulate the functions of dendritic cells (DCs) through multiple modes of actions, such as direct cell-cell contact and paracrine secretion of various soluble factors, e.g., prostaglandin E2 (PGE2) [29, 30], leading to phenotype changes and inhibition of DC maturation and, consequently, reduced antigen presentation capacity [31–33] and increased T cell tolerance [34]. With regard to mast cells, several lines of evidence have shown that MSCs inhibit their proliferation and degranulation-mediated activation [35–37] through the secretion of PGE2 [30]. With regard to neutrophils, MSCs inhibited their apoptosis and respiratory burst cycle through the secretion of IL-6, IFN-*α*, G-CSF, and TGF-*β* [29, 38]. Meanwhile, MSCs have been shown to inhibit infiltration, activation, and chemotaxis of neutrophils as evidenced by reduced secretion of chemokines and proinflammatory cytokines, e.g., C5a (a keratinocyte chemoattractant), MIP-2, fractalkine, LTB4, IFN-*γ*, and CXC chemokines [39, 40].

Among adaptive immune cells, MSCs could inhibit immune responses and proliferation of T cells either through direct cell-cell contact or through secretion of a large panel of soluble factors, such as HGF, TGF-*β*1, PGE2, and IDO [23]. MSCs have potent inhibitory effects on activation of different subtypes of T helper cells, such as Th1 and Th17, as evidenced by reduced secretion of proinflammatory cytokines and chemokines, such as IL-1*α*, IL-1*β*, IFN-*γ*, CXCL9, TNF-*α*, CXCL10, and IL-17 [41, 42]. On the other hand, MSCs can promote the function of Th2 cells and regulatory T cells (Tregs) as evidenced by increased production of Th2 and anti-inflammatory cytokines, e.g., IL-3, IL-5, IL-10, IL-13, and the Th2 chemokine I-309 [41, 43–45]. Lastly, MSCs significantly suppressed the proliferation and maturation of B cells through the secretion of COX-2-dependent PGE2 [37].

## 4. Trophic Paracrine Effects of MSCs

Accumulating evidence supports the notion that MSCs exert their therapeutic effects under various pathological settings through their paracrine secretion of a panel of trophic factors with a wide range of biological functions (Figure 1(c)). A set of factors produced by MSCs, such as HGF, TGF-*β*, PGE2, IDO, IL-10, TSG-6, and human leukocyte antigen-G5, are involved in MSC-mediated immunosuppressive and anti-inflammatory functions [46]. Of note, certain proinflammatory cytokines produced by activated immune cells, e.g., IFN-*γ* and TNF-*α*, can stimulate the production of immunosuppressive mediators IDO and iNOS/NO in MSCs, which subsequently contribute to MSC-mediated immunosuppressive and anti-inflammatory functions [5, 47]. In addition, some of the soluble factors secreted by MSCs, such as MIP-1 and MCP-5, could promote the migration of fibroblasts [48], keratinocytes, endothelial cells, and macrophages [49], thus contributing to tissue repair and regeneration. On the other hand, some trophic factors secreted by MSCs, such as EGF, IGF, KGF, IGFBP-1, and VEGF, have proproliferative and antiapoptotic effects on various types of host tissue cells such as keratinocytes, hair follicle cells, and endothelial cells [49], while some other secreted factors by MSCs, such as MMPs, Ang-1, VEGF, serine proteases, and serine protease inhibitors, have proangiogenesis, antifibrosis, and antioxidant functions, all of which are beneficial to tissue regeneration [25, 50–54]. Taken together, MSCs can interact with various types of host cells through their paracrine secretion of a myriad of bioactive soluble factors, thus contributing to the establishment of a proregenerative microenvironment favorable to tissue regeneration and homeostasis.

## 5. MSC-Released Extracellular Vesicles

Cells release extracellular vesicles (EVs) as one of the intercellular communication platforms. EVs could be mostly classified based on their diameter, including apoptotic bodies (1-5 *μ*m), microvesicles (MVs; 500-1000 nm), and exosomes (30-200 nm). EVs are widely distributed in body fluid and secreted in cell culture supernatants, which are rich in lipids, proteins, nucleic acids, and other components. Exosomes are released after fusion with the plasma membrane through forming multivesicular bodies, while MVs are released by directly shedding from the plasma membrane [5] (Figure 1(c)). In the following text, we collectively refer to exosomes and MVs as EVs.

In recent years, a growing body of evidence has shown that MSC-released EVs exhibit potent regulatory effects on various types of cells through transferring bioactive components to the recipient target cells. For example, MSC-EVs exert anti-inflammatory effects on inflammatory immune cells, such as M1 macrophages, DCs, and CD4+ Th1 and Th17 cells, through the intercellular transfer of immunoregulatory miRNAs and bioactive proteins, thus making them undergo phenotypic conversion into anti-inflammatory M2 macrophages, regulatory DCs, and Tregs. Additionally, MSC-EVs can promote tissue repair and regeneration through promoting the survival, proliferation, and regenerative potential of other types of resident cells. For instance, Wen et al. [55] showed that MSC-EVs inhibited hypoxia-induced apoptotic damages of cardiomyocytes by transferring miR-144 to target the PTEN/AKT pathway that plays an important role in protecting cardiomyocytes from ischemic/hypoxic damages. Moon et al. [56] found that MSC-EVs significantly mitigated stroke, possibly by promoting neurogenesis and angiogenesis by transfer of miR-184 and miR-210. Yan et al. [57] reported that MSC-EVs promoted the recovery of hepatocytes from oxidative stress-induced injuries through transferring GPX1 to suppress oxidative stress-induced apoptosis. Li et al. [58] investigated that MSC-EVs alleviated irradiation- (IR-) induced lung injury, possibly by inhibiting both the intrinsic and extrinsic apoptotic pathways in lung epithelial cells through the relay of miR-21-5p. Taken together, these findings suggest that MSC-released EVs exert therapeutic effects on various pathological conditions that are comparable to those conferred by MSC transplantation, thus serving as potential cell-free products for regenerative therapy of a variety of diseases.

## 6. Applications of MSCs in Cutaneous Diseases

Due to the potent regenerative potentials and trophic paracrine effects, MSCs and their derivative products are emerging as promising therapeutics for a spectrum of diseases, including inflammatory skin disorders [12]. Through searching the ClinicalTrials.gov database, 67 clinical trials on MSC-based therapies for cutaneous diseases were identified (Table 1), which were described in detail in the following sections.

### 6.1. Vitiligo

Vitiligo and alopecia areata (AA) are common chronic and recurrent autoimmune skin disorders characterized by white spots on the skin (vitiligo) and bald spots on the scalp (AA) because of selective destruction of melanocytes (MC) [2, 59, 60]. Most recently, Zhu et al. [60] found that vitiligo patients present with a high level of PTEN expression that may contribute to the impairment of melanocytes. In addition, it has been shown that MSCs could promote cell proliferation and suppress oxidative stress-induced apoptosis in human melanocytes by targeted inhibition of the PTEN/PI3K/AKT pathway, which may contribute to the therapeutic effects of MSCs on vitiligo. In a recent study, 23 vitiligo patients were treated by transplantation of autologous melanocytes, which showed that the transplantation efficiency of autologous melanocytes might be predicted by perilesional infiltration of CD8+ T cell activities [61]. Meanwhile, the results showed that dermal MSCs (DMSCs) could significantly inhibit the skin-homing activity of CD8+ T lymphocytes, suggesting that DMSCs might be concomitantly applied to improve the transplantation efficiency and therapeutic efficacy of autologous melanocytes in treating vitiligo [61].

### 6.2. Melanoma

Melanoma is the most malignant skin cancer, a process characterized by a linear transformation progression of normal melanocytes through various precursor lesions and ultimately to melanoma [62]. Several lines of evidence have shown that MSCs can be utilized as cellular vehicles to suppress the growth of melanoma through delivering IFN-*β* in the tumor microenvironment [63–65]. Wang et al. [66] reported that BMSCs transduced with pAd5-CMV-CYP2E1 recombinant adenovirus can act as an intermediate carrier to promote the killing effect of bystanders on melanoma cells in vitro and suppress the growth of cancer cells by activating 5-(3,3-dimethyl-1-triazeno)imidazole-4-carboxamide in an established mouse model of human melanoma. In addition, CM-FDMSC showed inhibitory effects on the tumorigenesis of A375 melanoma cells through promoting apoptosis, possibly by interfering with PI3K/AKT and mitogen-activated protein kinase (MAPK) signaling pathways, and a reduced BCL-2/BAX ratio [67]. Notwithstanding, some studies have shown that MSCs possess protective effects on melanoma cells. For instance, ADSCs could support proliferation and inhibit apoptosis and the response of melanoma cells to cytotoxic drugs in vitro. Meanwhile, ADSC-secreted soluble factors, e.g., VEGF, G-CSF, and SDF-1alpha/CXCR4, synergistically contribute to the formation of a proinflammatory tumor microenvironment, thus facilitating tumor growth [68]. Most recently, a study showed that genetically modified murine ADSCs producing IL-2 favored B16F10 melanoma cell proliferation in an immunocompetent mouse model of subcutaneous and lung metastatic melanoma [69].

### 6.3. Epidermolysis Bullosa

The successful use of hematopoietic cell transplantation (HCT) has previously been shown to treat pediatric patients with RDEB [70, 71], an intractable genetic blistering skin disease caused by mutations to the COL7A1 gene that deactivated the production of functional type VII collagen protein (C7) essential for skin integrity [72, 73]. Several lines of evidence have demonstrated that MSCs enhanced therapeutic effects of HCT graft on pediatric RDEB partly due to their intrinsic immunomodulatory, trophic properties and restorative effects on C7 production [71, 74–78]. Meanwhile, the tissue distress factor, high-mobility group box-1 (HMGB1) [79], Ccl27-Ccr10 chemotactic axis [80], and SDF-1*α*/CXCR4 signaling axis [72] have been shown to promote the recruitment of endogenous MSCs to skin lesions, thus attenuating the pathology of epidermolysis bullosa. Several clinical trials indicated that intradermal or intravenous administration of allogeneic MSCs showed therapeutic effects on recessive dystrophic epidermolysis bullosa [81, 82]. Currently, there are eight registered clinical trials on MSC-based therapy of epidermolysis bullosa as listed in the ClinicalTrials.gov (Table 1).

### 6.4. Photoaging

Photoaging refers to skin aging associated with ultraviolet radiation (UVR) exposure [83]. Jeong et al. [84–87] reported the successful use of MSCs and MSC-derived condition medium (CM) to improve wrinkling and reduce pigmentation on the photoaged skin in a mouse model through stimulating the expression of collagen and TGF-*β*, increasing dermal thickness, and decreasing MMP and IL-6 expression [48, 85, 87–89]. Additionally, MSCs and MSC-CM could decrease UVB-induced apoptotic cell death, upregulate antioxidant response element (ARE), increase SOD and GSH-Px activities, and attenuate the upregulation of malonaldehyde [85, 89, 90], all of which are beneficial to confining the photoaging process. Currently, there is one ongoing clinical trial on MSC-based therapy of photoaging (NCT01771679).

### 6.5. Psoriasis

Psoriasis is a T cell-mediated inflammatory autoimmune disease [91], characterized by an imbalance between the Th1/Th17 and Th2 cytokines [92]. Campanati et al. [93, 94] showed that MSCs isolated from psoriatic skin lesions exhibited compromised ability to inhibit T cell proliferation and activation, which might be attributed to their decreased capacity of secreting cytokines [94, 95]. Further studies indicated that the microenvironment in psoriasis could promote the expression of proinflammatory and angiogenetic factors by MSCs, thus contributing to the development of psoriatic skin lesions [96]. On the other hand, MSCs could mitigate psoriatic skin lesions by reducing the local levels of angiogenic and proinflammatory mediators [92, 97] and inhibiting the inflammatory responses of keratinocytes [98] and activation and differentiation of DC-mediated CD4+ T cells [99]. MSCs from skin lesions of psoriatic patients could promote proliferation and inhibit apoptosis of keratinocytes, which not only result in abnormal thickening of the epidermis [100] but also lead to dermal microvasculature formation/angiogenesis through increasing the expression of EDIL3, AMOT, and ECM [101]. Early clinical studies have demonstrated the safety and tolerance in psoriatic patients following transplantation of autologous MSCs [102]. In addition, Chen et al. [103] documented that the treatment of two cases of patients with psoriasis vulgaris with UCB-MSCs led to 4 or 5 years of remission. These findings suggest that replacing the abnormal resident MSCs in psoriatic skin lesions with autologous MSCs or allogeneic MSCs from healthy donors is worthy of further clinical studies on a large scale to develop safe and effective MSC-based therapy for psoriasis [104]. Currently, there are nine clinical trials on MSC-based therapy of psoriasis as listed in the ClinicalTrials.gov (Table 1).

### 6.6. Systemic Autoimmune Disease-Associated Skin Manifestations

Due to their potent immunosuppressive and anti-inflammatory properties, MSCs have been extensively explored as promising therapeutics for autoimmune skin diseases, such as dermatomyositis (DM) and alopecia areata (AA), and various systemic autoimmune/autoinflammatory diseases, such as systemic sclerosis (SSc), systemic lupus erythematosus (SLE), and graft versus host disease (GVHD) involved with severe skin manifestations (AA) [105–113]. Numerous clinical trials have been conducted to evaluate the therapeutic efficacy of MSCs in treating autoimmune disease-associated skin manifestations. For instance, Guiducci et al. [114, 115] reported that intravenous infusion of autologous MSCs improved vascularization, restored blood flow, and reduced skin necrosis in SSc patients. Liu et al. [116] reported that systemic infusion of MSCs significantly mitigated the severity of SLE as evidenced by attenuated proteinuria and hypocomplementemia. In addition, Sun et al. [117] reported that the disease activity index of SLE patients reduced by half within 6 months following systemic infusion of allogeneic MSCs from HLA-disparate family members. For GVHD patients, studies showed that HCT, together with the systemic infusion of MSCs after myeloablation, significantly reduced the incidence rate of acute and chronic GVHD at 6 months posttransplantation [118]. In addition, transplantation of allogeneic MSCs appears to be safe and effective in treating drug-resistant DM patients [113]. Regarding the treatment of AA, a few clinical trials showed that administration of ADSC-CM or ADSCs could increase hair density and thickness [119]. Currently, there are seven clinical trials on MSC-based therapy of systemic sclerosis as listed in the ClinicalTrials.gov (Table 1).

### 6.7. Cutaneous Ulcers

The therapeutic effects of MSCs on chronic cutaneous ulcers, such as pressure ulcers, radiation skin ulcers, diabetic skin ulcers, and leprosy skin ulcers, have been evaluated in both preclinical studies and clinical trials [120]. With regard to the treatment of pressure ulcers, MSCs promote ulcer healing in mice probably due to the reduced oxidative stress-mediated cellular apoptosis, vascular damages, and ER stress [121] and the induction of adipogenic differentiation and regeneration of the underlying architecture of the skin [122]. Of note, a recent study showed that transplantation of MSCs failed to promote pressure ulcer healing, possibly because of their transition retention and marginal differentiation capacity into tissue-specific cells [123]. With regard to diabetic skin ulcers, transplantation of allogeneic MSCs would improve the healing of diabetic skin ulcers by augmenting angiogenesis in a diabetic rabbit ear ulcer model [124]. Meanwhile, in a diabetic rat ulcer model, intramuscular transplantation of BMSCs showed increased survival ability and promoted the expression level of VEGF in the wound tissues at the later stage as compared to subcutaneously transplanted BMSCs [125]. In a radiation-induced skin ulcer model in rats, treatment with MSC-CM accelerated wound closure and healing, possibly by promoting angiogenesis and regeneration of sebaceous glands [126]. Furthermore, the therapeutic effects of MSCs on chronic skin ulcers have been reported in several clinical studies [120, 127]. For instance, in a study with 2 patients with radiation-induced skin ulcers, transplantation of autologous MSCs achieved complete epithelialization of the ulcer surface [128]. Mechanistically, MSCs promote the healing of radiation-induced skin ulcers through facilitating neovascularization and reepithelization due to the activation of the PI3K/AKT signaling pathway [129]. In another study with 22 patients with chronic plantar ulcers in leprosy, 21 patients showed improvement in ulcerous lesions following treatment with hAMMSC-CM [130]. Lastly, in one study with 53 patients with severe symptoms of Fontaine's II-IV diabetic foot ulcers together with varying degrees of lower extremity arterial abnormalities, transplantation of hUCB-MSCs after angioplasty increased neovascularization accompanied by complete or progressive healing of ulcers [131]. Currently, there are thirty-one registered clinical trials on MSC-based therapy of skin ulcers, among which twenty-six clinical trials on diabetic foot ulcers (Table 1).

### 6.8. Atopic Dermatitis

Atopic dermatitis (AD) is a typical abnormal T cell-mediated immune disorder characterized by a significant imbalance between Th2 and Th1/Th17, particularly in the early phase, whereas a mixed Th1/Th2 pattern appears in the chronic stage [132]. MSCs from the skin of AD patients showed an upregulation of a panel of Th1/Th17 cytokines, while Th2 cytokines were downregulated, suggesting that dysregulation of MSCs might also be involved in the pathogenesis of AD [132]. In a contact hypersensitivity (CHS) model in mice, MSCs in adipose tissues (ADSCs) may contribute to the self-limiting course of allergic contact dermatitis (ACD) by decreasing the expression of IFN-*γ* rather than increasing the expression of IL-10 [133]. On the other hand, treatment with exogenous ADSCs improved AD by decreasing the expression levels of cytokines and chemokines, such as IL-5, MIP-1ss, MIP-2, CCL5, and IL-17 [134]. Another study showed that allogeneic and syngeneic clonal BMSCs exhibited therapeutic effects on AD, possibly by suppressing T cell and B cell functions, decreasing the serum IgE level, and inhibiting cell infiltration in skin lesions and the expression of IL-4 in the lymph node and skin [135]. Most recently, it has been shown that MSC-derived EVs exerted potent therapeutic effects on AD in mice as evidenced by the improvement in pathological symptoms/clinical scores, decreased serum IgE level and number of eosinophils in the blood, and reduced infiltration of mast cells, CD86+ and CD206+ cells, and inflammatory cytokine levels in AD skin lesions [136, 137]. In a clinical trial, the safety and efficacy of hUCB-MSCs have been validated in the treatment of moderate-to-severe atopic dermatitis [138]. Currently, there are four registered clinical trials on MSC-based therapy of atopic dermatitis as listed in the ClinicalTrials.gov (Table 1).

### 6.9. Skin Wounds and Burns

Wound healing is a complex process involving multiple layers of integrated interactions among various cell types and bioactive molecules, whereas any aberrant change in this process can lead to compromised wound healing, such as abnormal scar formation [139]. In the last two decades, MSCs of diverse tissue origins have been extensively explored as a potential regenerative therapy to facilitate normal and abnormal skin wound healing through multiple modes of actions, such as promoting resolution of the inflammation, vascularization, migration, and proliferation of epithelial cells, matrix remodeling, and inhibition of apoptosis [139, 140]. For example, transplantation of autologous MSCs led to almost complete healing in the skin wound of pigs [141]. Zhang et al. showed that systemic administration of human gingiva-derived MSCs (GMSCs) facilitated full-thickness skin wound healing in mice through promoting reepithelialization, angiogenesis, and regenerative M2 macrophage polarization [27]. In the skin wound model of mice and rabbits, xenotransplantation of MSCs also promoted skin wound healing but did not cause any immunologic responses [142, 143]. In addition, a study showed that intradermal application of MSCs accelerated full-thickness skin wound healing in Albino rats [144]. In a rat diabetic skin wound model, transplanted MSCs could survive in the wounds and promote wound healing through angiogenesis [145]. In clinical studies, transplantation of MSCs could effectively promote the healing of skin burn wounds by regenerating functional sweat glands [146]. Clinical trials have also been conducted using autologous and allogeneic MSCs derived from adipose tissue and bone marrow to treat skin wounds and burns [147–149]. Most recently, the MSC secretome, including MSC-CM and MSC-derived EVs, has been shown to promote skin wound healing through multiple functions, e.g., regulating fibroblast functions/matrix remodeling and promoting reepithelialization, angiogenesis, resident cell proliferation, and polarization of regenerative M2 macrophages [48, 54, 150–154]. In addition, MSCs have been transplanted in combination with different types of biomaterials, such as the collagen-chitosan laser drilling acellular dermal matrix, microspheres, R120 nanofiber, graphene, silk fibroin, PVA, PLGA, and hydrogel, which improved the local retention and proliferation of transplanted MSCs and showed promising therapeutic effects on skin wound healing [155–163].

### 6.10. Keloids and Scars

Keloids are a skin disorder characterized by excessive collagen deposition into the extracellular matrix (ECM), while its pathogenesis remains largely unknown. Several lines of evidence have implied the potential role of MSCs with aberrant phenotypes and their special inflammatory niche in the keloid pathogenesis [164–166]. Even though there is still a lack of an animal model for human keloids, several in vitro studies have shown that human MSCs of different tissue origins, such as ADSCs, BMSCs, amnion-derived MSCs, fetal dermal MSCs, and human Wharton's jelly (umbilical cord) MSCs, had obvious paracrine inhibitory effects on the proliferation, profibrotic phenotype, the production of the extracellular matrix (ECM), and other bioactivities of fibroblasts derived from human keloids and hypertrophic scars [167–174]. In rabbit ear skin wound models, transplantation of autologous MSCs showed preventive effects on hypertrophic scar formation involving multiple potential mechanisms such as inhibition of the proliferation and transformation of fibroblasts into myofibroblasts; decreased expression of TGF-*β*1, type I and type III collagens, and inflammatory responses; and increased expression of decorin [175–177]. Recent studies indicated that the application of MSC-derived exosomes could also prevent scar formation through horizontal transfer of miRNAs to suppress differentiation of fibroblasts into myofibroblasts [178, 179]. Yates et al. [180] showed that cotransplantation of MSCs with fibroblasts could normalize matrix production, thus attenuating hypertrophic scarring. In addition, BMSCs genetically modified to overexpress TGF-*β*3 showed obvious effects to reduce the formation of scar tissue in a rabbit skin wound model [181]. Most recently, clinical trials have been performed to evaluate the antiscarring effects of MSCs on Cesarean section skin scars or acne scar formation [182, 183].

## 7. Challenges and Perspectives

To date, a growing body of preclinical and clinical studies has demonstrated the beneficial effect of MSC-based therapy for a wide spectrum of diseases, including various autoimmune and inflammatory skin disorders. However, there are several major challenges faced in the clinical translation of MSC-based regenerative therapies. One of the major challenges might be the large variations in the therapeutic efficacy of MSCs due to their heterogeneity caused by various intrinsic and extrinsic factors. Intrinsically, the different tissue origins, age, and health status (niche factors) can affect the property and function of MSCs. Extrinsically, the isolation, culture, and ex vivo expansion conditions can also affect the property and biological functions of MSCs [184]. Therefore, it would be critical to identify the appropriate donors and tissue sources of MSCs and optimize the isolation and ex vivo expansion conditions so as to obtain scalable MSC products with consistent quantity and quality. Recently, microfluidic single-cell characterization, an emerging technique, has offered particularly dramatic strategies for identifying and isolating the most effective cells for therapeutic use [185]. During the downstream application process, many factors such as the dosage, the route, the timing, and the frequency of cell delivery might significantly affect the therapeutic efficacy of MSC-based therapy [185]. Another concern is the safety of MSCs, particularly their tumorigenic potentials, following the long-term transplantation. To date, there is still no efficient way to follow up the fate and behavior MSCs following transplantation in vivo. Recently, a case study reported tumor formation at the site of spinal injury of a patient following local transplantation of adult olfactory mucosal cells [186]. Most recently, several studies have described the side effects of MSC therapy in different diseases, including graft versus host disease and cardiac, neurological, and orthopedic disorders [187].

Accumulating evidence has shown that MSC-derived EVs exhibited potent immunomodulatory/anti-inflammatory and pleiotropic effects as the parental MSCs did. More recently, some preclinical and early clinical studies have shown that MSC-EVs displayed therapeutic effects on several disease models, including cutaneous diseases [85, 119, 130, 136, 137]. Therefore, MSC-EVs hold great promises to be developed as potential cell-free therapeutic products that can avoid the major challenges faced in the use of MSCs [188].

## 8. Conclusions

In the last two decades, much progress has been made in delineating the molecular mechanisms of action of MSCs and their potential application in regenerative therapy for a wide spectrum of diseases, including various autoimmune and inflammatory skin disorders. To date, accumulating evidence supports the notion that MSCs exert their therapeutic effects under various pathological settings through multiple modes of functions mediated by their paracrine secretome containing a myriad of bioactive factors, including extracellular vesicles (EVs). However, much effort is still required to further investigate the specific cellular and molecular mechanisms by which MSCs of different tissue origins and their cell-free derivative products exert their unique therapeutic effects on certain cutaneous diseases.

## Figures and Tables

**Table 1:** MSC-based clinical trials for cutaneous diseases.

ClinicalTrials.gov identifier	Phase/status/(start dates)	Conditions	Type of cells	Outcome	Country
NCT02824393	Early phase 1 Completed (March 2017)	Urticaria	Autologous MSCs	MSC therapy more effective than conventional treatment, resulted in longer recovery	Turkey
NCT01771679	Phase 1 Phase 2 Suspended (July 2015)	Photoaging	Allogeneic BMSCs	Ongoing	United States
NCT03765957	Early phase 1 Not yet recruiting (February 2019)	Psoriasis	MSCs	Ongoing	China
NCT02304562	Phase 1 Unknown (January 2013)	Sweat gland diseases	UCB-MSCs	Ongoing	China
NCT02491658	Phase 1 Phase 2 Unknown (April 2015)	Psoriasis vulgaris	UC-MSCs	Ongoing	China
NCT02213705	Phase 1 Phase 2 Unknown (May 2014)	Systemic scleroderma	Allogeneic MSCs	Ongoing	France
NCT03745417	Phase 1 Phase 2 Recruiting (August 2018)	Psoriasis	UC-MSCs	Ongoing	China
NCT03265613	Phase 1 Phase 2 Active, not recruiting (September 2017)	Psoriasis	ADSCs	Ongoing	China
NCT03564808	Phase 2 Recruiting (June 2018)	Skin pigmentation over contour deformities of the face, trauma, Romberg's disease	MSCs	Ongoing	Pakistan
NCT03392311	Phase 1 Phase 2 Recruiting (August 2019)	Psoriasis	ADSCs	Ongoing	China
NCT03424629	Phase 1 Unknown (June 2018)	Moderate and severe plaque psoriasis	UC-MSCs	Ongoing	China
NCT00962923	Phase 1 Phase 2 Unknown (August 2009)	Systemic sclerosis	Allogeneic MSCs	Ongoing	China
NCT04275024	Not applicable Recruiting (April 2020)	Psoriasis	ADSCs	Ongoing	China
NCT02034786	Phase 1 Unknown (March 2015)	Aesthetics procedure	Autologous MSCs	Ongoing	Brazil
NCT02685722	Phase 1 Completed (January 2012)	Skin ulcers	UC-MSCs	—	China
NCT04356287	Phase 1 Phase 2 Not yet recruiting (October 2020)	Systemic sclerosis	UC-MSCs	Ongoing	—
NCT04520022	Phase 1 Phase 2 Completed (October 2016)	Recessive dystrophic epidermolysis bullosa	UC-MSCs	—	Korea
NCT02975960	Not applicable Completed (October 2016)	Systemic sclerosis	Autologous ADSCs	—	Korea
NCT04153630	Phase 1 Phase 2 Active, not recruiting (May 2018)	Recessive dystrophic epidermolysis bullosa	BMSCs	Ongoing	Spain
NCT03529877	Phase 1 Phase 2 Recruiting (January 2019)	Recessive dystrophic epidermolysis bullosa	Allogeneic ABCB5-positive MSCs	Ongoing	United States, Austria, France, German, Italy, United Kingdom
NCT02888704	Phase 1 Completed (July 2016)	Atopic dermatitis	MSCs	—	Korea
NCT03887208	Phase 1 Phase 2 Completed (January 2018)	Skin, scar, cutis laxa, keloid, cicatrix	Autologous ADSCs	—	Poland
NCT02494752	Not applicable Unknown (August 2015)	Romberg's disease, craniofacial microsomia, lipodystrophy, mixed connective tissue disease	Fat graft enriched with MSCs	Ongoing	United Kingdom
NCT01033552	Phase 2 Recruiting (January 2010)	Epidermolysis bullosa	MSCs	Ongoing	United States
NCT02582775	Phase 2 Recruiting (March 2016)	Epidermolysis bullosa	Allogeneic MSCs	Ongoing	United States
NCT02834858	Phase 1 Unknown (January 2016)	Peripheral vascular disease, ischemia, diabetic foot	UC-MSCs	Ongoing	China
NCT01216865	Phase 1 Phase 2 Unknown (January 2011)	Diabetic foot, critical limb ischemia	UC-MSCs	Ongoing	China
NCT04464213	Phase 1 Not yet recruiting (August 2020)	Diabetic foot ulcer	pMSCs	Ongoing	—
NCT04432545	— Available (-)	Diffuse cutaneous systemic sclerosis with refractory pulmonary involvement	Allogeneic MSCs	Ongoing	China
NCT01932021	Not applicable Unknown (April 2013)	Skin ulcer	ADSCs	Ongoing	France
NCT02304588	Phase 1 Unknown (January 2013)	Diabetic foot, lower limb ischemia	MSCs	Ongoing	China
NCT03259217	Phase 1 Unknown (October 2017)	Diabetic foot ulcers	MSCs seeded in chitosan scaffold	Ongoing	Egypt
NCT04104451	Phase 1 Recruiting (November 2019)	Diabetic foot ulcer	CLMSCs	Ongoing	United States
NCT00955669	Phase 1 Completed (August 2009)	Diabetic critical limb ischemia and foot ulcer	Autologous BMSCs	Effective treatment and no recurrence in the next 10-year follow-up span	China
NCT04466007	Phase 2 Not yet recruiting (October 2020)	Diabetic foot with critical limb ischemia	Allogeneic ADSCs	Ongoing	—
NCT03676400	Not applicable Completed (October 2018)	Androgenic alopecia	UC-MSCs	—	Korea
NCT02672280	Phase 1 Phase 2 Unknown (May 2016)	Wounds, diabetic foot ulcers, burns	Medical collagen membrane with MSCs	Ongoing	China
NCT03865394	Phase 1 Phase 2 Recruiting (September 2018)	Diabetic foot ulcer	Autologous ADSCs	Ongoing	Poland
NCT04497805	Phase 2 Not yet recruiting (August 2020)	Diabetic foot ulcer	Allogeneic ADSCs	Ongoing	—
NCT03013049	Not applicable Unknown (January 2016)	Vitiligo	DMSCs	Ongoing	India
NCT02579369	Phase 1 Phase 2 Unknown (October 2015)	Dystrophic epidermolysis bullosa	Allogeneic MSCs	Ongoing	Korea
NCT02619877	Phase 2 Completed (October 2015)	Diabetic foot ulcer	Allogeneic ADSCs	Complete wound healing in the majority of patients	Korea
NCT02796079	Phase 1 Unknown (January 2015)	Peripheral vascular disease, ischemia, diabetic foot	MSCs	Ongoing	China
NCT03370874	Phase 3 Unknown (July 2018)	Diabetic foot ulcer	Allogeneic ADSCs	Ongoing	Korea
NCT03183726	— Completed (January 2016)	Diabetic foot ulcer	Allogeneic ADSCs	—	Korea
NCT03754465	Phase 2 Recruiting (November 2018)	Diabetic foot ulcer	Allogeneic ADSCs	Ongoing	United States
NCT03629002	— Unknown (September 2018)	Systemic scleroderma	MSCs	Ongoing	France
NCT03060551	Early phase 1 Completed (July 2018)	Systemic sclerosis	Autologous ADSCs	Improvement of skin fibrosis, hand edema, active ulcers, and quality of life	Korea
NCT03183804	— Unknown (June 12, 2017)	Diabetic foot ulcer	Allogeneic ADSCs	Ongoing	Korea
NCT03248466	Early phase 1 Recruiting (August 2017)	Diabetic foot ulcer	BMSCs	Ongoing	China
NCT03257098	Phase 1 Phase 2 Recruiting (November 2017)	Skin ulcer venous stasis chronic	Allogeneic ABCB5-positive MSCs	Ongoing	Germany
NCT03252340	— Active, not recruiting (September 2017)	Atopic dermatitis	MSCs	Ongoing	Korea
NCT02918123	Phase 1 Recruiting (January 2018)	Psoriasis	Allogeneic UCB-MSCs	Ongoing	Korea
NCT03183934	— Unknown (July 2017)	Dystrophic epidermolysis bullosa	Allogeneic ADSCs	Ongoing	Korea
NCT04173650	Phase 1 Phase 2 Not yet recruiting (September 2020)	Dystrophic epidermolysis bullosa	MSC-EVs	Ongoing	—
NCT03211793	Phase 1 Phase 2 Unknown (November 2018)	Systemic sclerosis Digital ulcer	MSCs	Ongoing	Netherlands
NCT01065337	Phase 2 Completed (August 2005)	Diabetic foot	BMSCs	Improvement of microcirculation and complete wound healing in the majority of patients	Germany
NCT01686139	Phase 1 Unknown (March 2016)	Diabetic foot ulcers	MSCs	Ongoing	Israel
NCT04179760	Phase 1 Phase 2 Recruiting (March 2020)	Atopic dermatitis	Allogeneic BMSCs	Ongoing	Korea
NCT02394886	Phase 1 Completed (November 2014)	Diabetic foot ulcer	Allogeneic ADSCs	—	Korea
NCT028310752	Phase 1 Unknown (January 2015)	Diabetic foot	ADSCs	Ongoing	China
NCT04569409	Phase 3 Recruiting (July 2020)	Diabetic foot ulcer	Allogeneic ADSCs	Ongoing	Korea
NCT03276312	Not applicable Completed (April 2015)	Diabetic foot	Autologous ADSCs	Improvement of healing rate	Italy
NCT00815217	Not applicable Unknown (February 2009)	Diabetic wounds	Autologous ADSCs	Ongoing	United States
NCT04137562	Phase 2 Recruiting (December 2019)	Atopic dermatitis	ADSCs	Ongoing	Korea
NCT02742844	Phase 1 Phase 2 Terminated (August 2016)	Skin ulcer venous stasis chronic	ABCB5-positive MSCs	Ongoing	Germany
NCT02619734	Phase 1 Unknown (August 2006)	Chronic leg ulcer Sickle cell disease	Autologous BMSCs	Ongoing	—
